# αGlcNAc and its catalyst α4GnT are diagnostic and prognostic markers in uterine cervical tumor, gastric type

**DOI:** 10.1038/s41598-019-49376-7

**Published:** 2019-09-10

**Authors:** Koichi Ida, Kazuhiro Yamanoi, Shiho Asaka, Hodaka Takeuchi, Tsutomu Miyamoto, Tanri Shiozawa, Jun Nakayama

**Affiliations:** 1 Department of Obstetrics and Gynecology, Shinshu University School of Medicine, Matsumoto, 390-8621 Japan; 2 Department of Molecular Pathology, Shinshu University School of Medicine, Matsumoto, 390-8621 Japan; 3 Institute for Biomedical Sciences, Interdisciplinary Cluster for Cutting Edge Research, Shinshu University, Matsumoto, 390-8621 Japan; 4 Department of Pathology, Keio University School of Medicine, Tokyo, 160-8582 Japan; 5 Department of Clinical Laboratory, Shinshu University Hospital, Matsumoto, 390-8621 Japan

**Keywords:** Tumour biomarkers, Cervical cancer

## Abstract

Cervical adenocarcinoma, gastric type (GAS) is not associated with human papilloma virus (HPV) infection. GAS patients prognoses are significantly worse compared with cervical adenocarcinoma associated with HPV infection, as their tumors exhibit resistance to conventional chemotherapy and radiotherapy. GAS is often associated with lobular endocervical glandular hyperplasia (LEGH), which is regarded as a precursor to GAS in the latest WHO classification. Recently, we reported that a decrease in expression of terminal α1,4-linked *N*-acetylglucosamine (αGlcNAc) relative to that of MUC6 was already apparent in atypical LEGH in the LEGH-GAS sequence. Here, we analyzed expression of α1,4-*N*-acetylglucosaminyltransferase (α4GnT), the sole enzyme catalyzing αGlcNAc biosynthesis, and that of αGlcNAc and MUC6 in cases representing non-neoplastic endocervical gland (NNEG) (11 cases), LEGH (26 cases) and GAS (12 cases). α4GnT protein was detected in a “dot-like” pattern, indicating localization in the Golgi apparatus in all 26 LEGH cases and 5 of 12 GAS cases. α4GnT- and αGlcNAc-positive cells largely overlapped, suggesting that α4GnT gene expression regulates αGlcNAc biosynthesis. Interestingly, all NNEG cases were negative for α4GnT and αGlcNAc expression, but 7 of 11 NNEG and all LEGH cases were MUC6-positive. In GAS cases, patients whose tumors were α4GnT- and αGlcNAc-positive had more favorable prognosis than others. Multivariate analysis revealed that positive expressions of α4GnT and αGlcNAc were independent prognostic indicators. These results indicate that α4GnT and αGlcNAc could serve as useful markers not only to distinguish LEGH from NNEG but to evaluate prognoses of GAS patients.

## Introduction

The prevalence of squamous cell carcinoma of the cervix has in past years significantly decreased in developed countries, whereas that of cervical adenocarcinoma has increased, particularly in young women^1–5^. The etiology of most cervical adenocarcinomas is infection with the high-risk, oncogenic human papillomavirus (HPV)^6,7^. This cervical carcinoma type was called usual endocervical adenocarcinoma (UEA). Fortunately, an HPV DNA test is highly sensitive, and UEA are found to be HPV-positive^8,9^. In the remaining non-HPV-associated adenocarcinomas, gastric-type adenocarcinoma (GAS) is the most common histologic subtype^8,10–12^. GAS prognosis is significantly worse than that of UEA: overall 5-year disease-specific survival rate of GAS is reportedly 30–42% in comparison with 74–91% for UEA, as GAS is more resistant to conventional chemotherapy and radiotherapy^13,14^.

In 1999, lobular endocervical glandular hyperplasia (LEGH) was reported as a distinct glandular lesion of the uterine cervix^15^. Interestingly, LEGH lesions reportedly secrete gastric pyloric-type mucin^16,17^. Therefore, GAS and LEGH share common clinical and histologic features and belong to the same spectrum as neoplasms with gastric gland differentiation. Some GAS cases are reportedly associated with LEGH, and LEGH was defined as a putative GAS-precursor in the 2014 WHO classification^13,18–20^.

**Figure 1:**
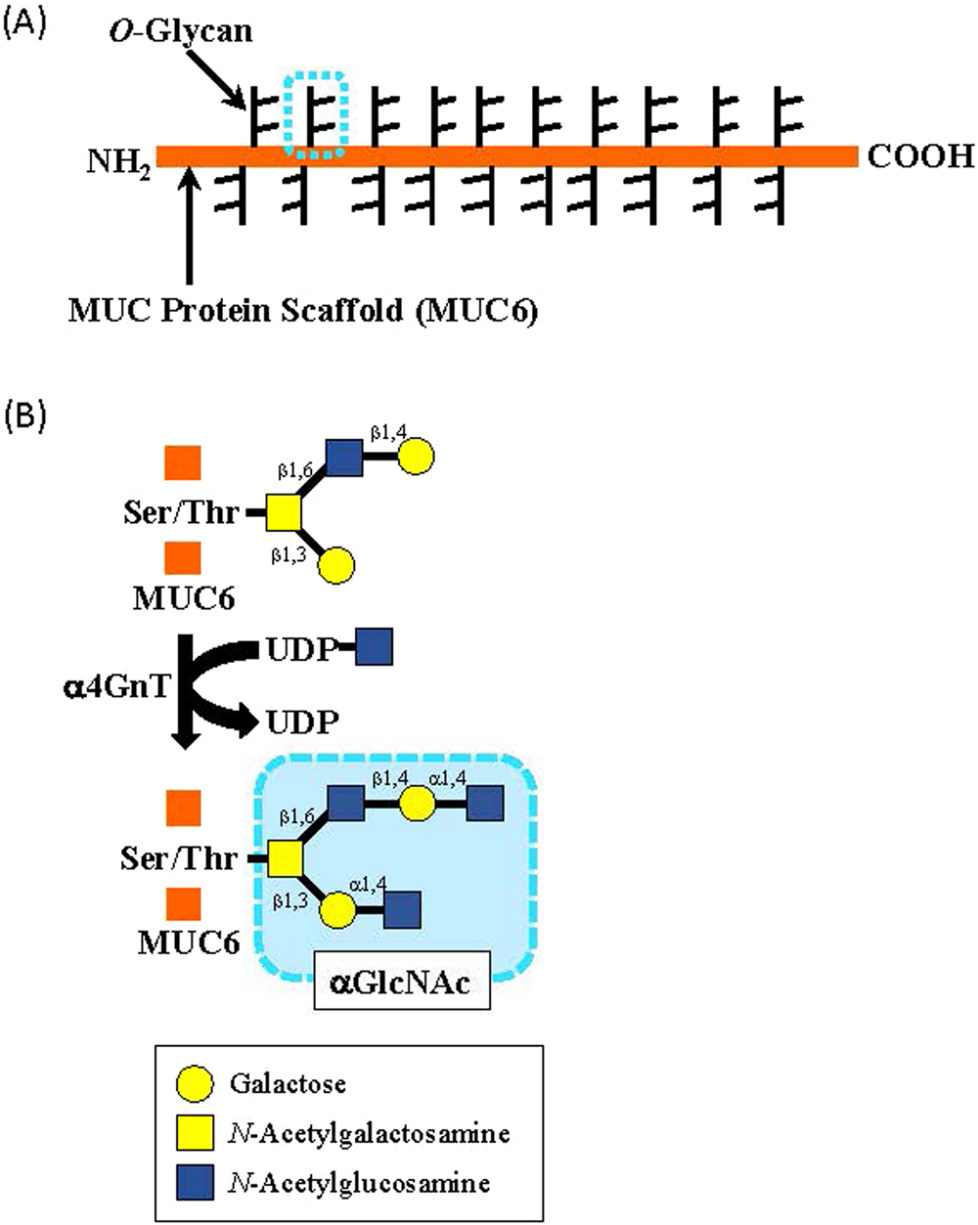
(**A**) Schema showing MUC6 scaffold and *O*-glycan residues. Blue dotted circle shows each *O*-glycan residue and corresponds to the blue dotted circle shown in B. (**B**) Schema showing αGlcNAc biosynthesis catalyzed by α4GnT, which transfers GlcNAc from UDP-GlcNAc to βGal residues of *O*-glycans linked to MUC6 Ser/Thr residues via an α1,4-linkage.

We previously analyzed alterations in specific sugar residues of gastric gland mucin in relationship to cancer progression^21–25^. Gastric gland mucin contains *O*-linked oligosaccharides (*O*-glycans) with terminal α1,4-linked *N*-acetylglucosamine (αGlcNAc) residues attached largely to a MUC6 scaffold (Fig. 1)^21,24,25^. Previously, we isolated cDNA encoding α1,4-*N*-acetylglucosaminyltransferase (α4GnT), the enzyme catalyzing αGlcNAc biosynthesis (Fig. 1), and then generated *A4gnt*-deficient mice^26,27^. These mutant mice showed αGlcNAc loss in gastric gland mucin and naturally developed gastric adenocarcinoma through a hyperplasia-dysplasia-carcinoma sequence without *Helicobacter pylori* infection^28^. We also evaluated αGlcNAc expression in human gastric adenocarcinoma and pyloric gland adenoma, which precedes gastric adenocarcinoma, and observed frequent loss of αGlcNAc expression in MUC6-positive differentiated-type adenocarcinoma and high-grade pyloric gland adenoma^22,23,25^. These results indicate that αGlcNAc functions as a tumor suppressor in gastric cancer.

In concordant with the idea, we evaluated that αGlcNAc and MUC6 expression in gastric gland mucin-producing tumors arising in extra-gastric organs. In pancreas, we observed significantly decreased αGlcNAc expression relative to MUC6 not only in invasive carcinoma but in its pre-malignant lesions, intraductal papillary mucinous neoplasm and pancreatic intraepithelial neoplasia^25,29^. Furthermore, we recently reported that αGlcNAc and MUC6 are co-expressed in typical LEGH, but αGlcNAc expression is reduced relative to MUC6 in atypical LEGH and minimal deviation adenocarcinoma (MDA)^30^. These findings support the idea that reduced αGlcNAc expression relative to MUC6 marks progression from pre-malignant lesions to cancers showing pyloric gland phenotypes in stomach, pancreas and uterine cervix and that αGlcNAc functions as a tumor suppressor. However, the expression pattern of α4GnT, which is responsible for αGlcNAc biosynthesis, in tumors arising from uterine cervix has not been assessed, and αGlcNAc expression related to GAS prognosis is unclear. Furthermore, MUC6, αGlcNAc and α4GnT expression in non-neoplastic endocervical gland (NNEG) has not been fully characterized.

In this study, we extend our previous studies and perform immunohistochemical assessment of expression of gastric gland mucin-related markers, including α4GnT, as well as αGlcNAc and MUC6 in LEGH, GAS and NNEG lesions. We also analyze potential associations between marker expression and prognosis and clinicopathological factors relevant to GAS. We report that α4GnT and αGlcNAc could serve as useful markers not only to distinguish LEGH from NNEG but to estimate prognosis of GAS patients.

## Materials and Methods

### Patients and tissue samples

Ethical approval was granted by the Institutional Review Board at Shinshu University School of Medicine, Matsumoto, Japan in accordance with the Declaration of Helsinki (no. 3996). Informed written consent was received from all participants. All research was performed in accordance with the relevant guidelines and regulations. We performed a total 49 specimens, which based on the latest WHO classification represented 26 LEGH cases (age range, 32–62; median, 47), 12 GAS cases (age range, 41–77 years; median, 55), and 11 NNEG cases from patients with uterine corpus leiomyoma (age range, 38–54; median, 45)^18^. All 49 specimens were fixed in 10% buffered formalin and embedded in paraffin wax. H.E.-stained sections were assessed by light microscopy.

Information of each case clinicopathological factors was obtained through electronic medical records from hospital information system of the Shinshu University Hospital. Data included date at diagnosis, date of surgery, age, tumor histology, ascites cytology, regional lymph node, treatment and prognosis. Clinicopathological staging of 12 GAS patients was based on the International Federation of Gynecology and Obstetrics (FIGO) Staging System^31^. Regional lymph node dissection was performed in all but one patient, who could not be assessed for lymph node metastatic status. Survival of all 12 GAS patients was followed at Shinshu University Hospital. Overall survival (OS) period was defined as the length of the time during patient alive after surgical cancer resection. Progression free survival (PFS) period was defined as the length of time during a patient lives without cancer progression and/or recurrence after surgical cancer resection. Cancer progression and recurrence was diagnosed by clinicians on the basis of physical examination, imaging and scintigraphy.

### Immunohistochemistry

The following primary antibodies were used: anti-α4GnT (I17K, polyclonal, 1:100 dilution), anti-αGlcNAc (clone HIK1083, 1:15, Kantokagaku, Tokyo, Japan), anti-MUC6 (clone CLH5, 1:100, Novocastra, Newcastle, UK) and anti-p16 (clone G175–405, 1:50; BD Biosciences, Franklin Lakes, NJ, USA). The anti-α4GnT antibody was previously prepared in our laboratory, and its specificity was validated by Western blot analysis and immunocytochemistry using gastric cancer AGS-α4GnT cells stably expressing α4GnT and mock-transfected AGS cells^24^. Three-micrometer-thick sections were deparaffinized in xylene and rehydrated in ethanol. Endogenous peroxidase activity was quenched by soaking sections in absolute methanol containing 0.3% hydrogen peroxide for 10 min. For anti-MUC6 and anti-p16, antigens were retrieved by boiling sections in a microwave in 10 mM Tris/HCl buffer (pH 8.0) containing 1 mM EDTA for 15 min. Sections were then exposed to primary antibodies at room temperature for 60 min. After 30 min incubation of secondary antibody at room temperature, the color reaction was developed with 3′3-diaminobenzidine (Dojindo, Kumamoto, Japan). Negative controls were established by omitting primary antibodies from the procedure.

Sections were evaluated by K. Ida and K. Yamanoi. As for p16, lesions exhibiting diffuse nuclear staining of moderate or strong intensity in more than half of the cells were judged positive, as described^32^. Immunostaining for α4GnT was evaluated as positive when detected in the supranuclear region in a “dot-like” pattern^24^. αGlcNAc and MUC6 were evaluated based on cytosolic staining. Scoring of α4GnT, αGlcNAc and MUC6 expression was undertaken as follows. First, cases in which ≥10% of the total number of endocervical or tumor cells of each specimen were positively-stained were judged positive, as described previously^23,33^. Second, expression levels of α4GnT, αGlcNAc and MUC6 were further scored semi-quantitatively from 0 to 3: 0 (<10% positive cells), 1 (10–33% positive cells), 2 (34–66% positive cells), and 3 (≥67% positive cells), as described previously^23,33^.

### Statistical analysis

Correlation between each stage (NNEG, LEGH and GAS) and the number of positive cases was analyzed by Fisher’s exact probability test. Comparisons of semi-quantitative immunoreactivity scores of α4GnT, αGlcNAc or MUC6 at each stage were performed using the Kruskal-Wallis test with post-hoc pairwise comparison of subgroups. For GAS patients’ analysis, clinicopathological parameters were compared using the Fisher’s exact probability test. Survival curves were constructed using the Kaplan–Meier method, and the difference between curves was evaluated by a log–rank test. Univariate and multivariate analyses were performed using the Cox proportional hazards regression model. All data analyses were performed using the Software Package for the Social Sciences (SPSS version 25; IBM, Armonk, NY, USA). *P*-values < 0.05 were considered statistically significant.

### Compliance with ethical standards

This study was approved by the ethics committee of Shinshu University School of Medicine, Japan (project no. 3996 was approved on April 3, 2018).

## Results

### α4GnT, αGlcNAc and MUC6 expression in each histological type

**Figure 2:**
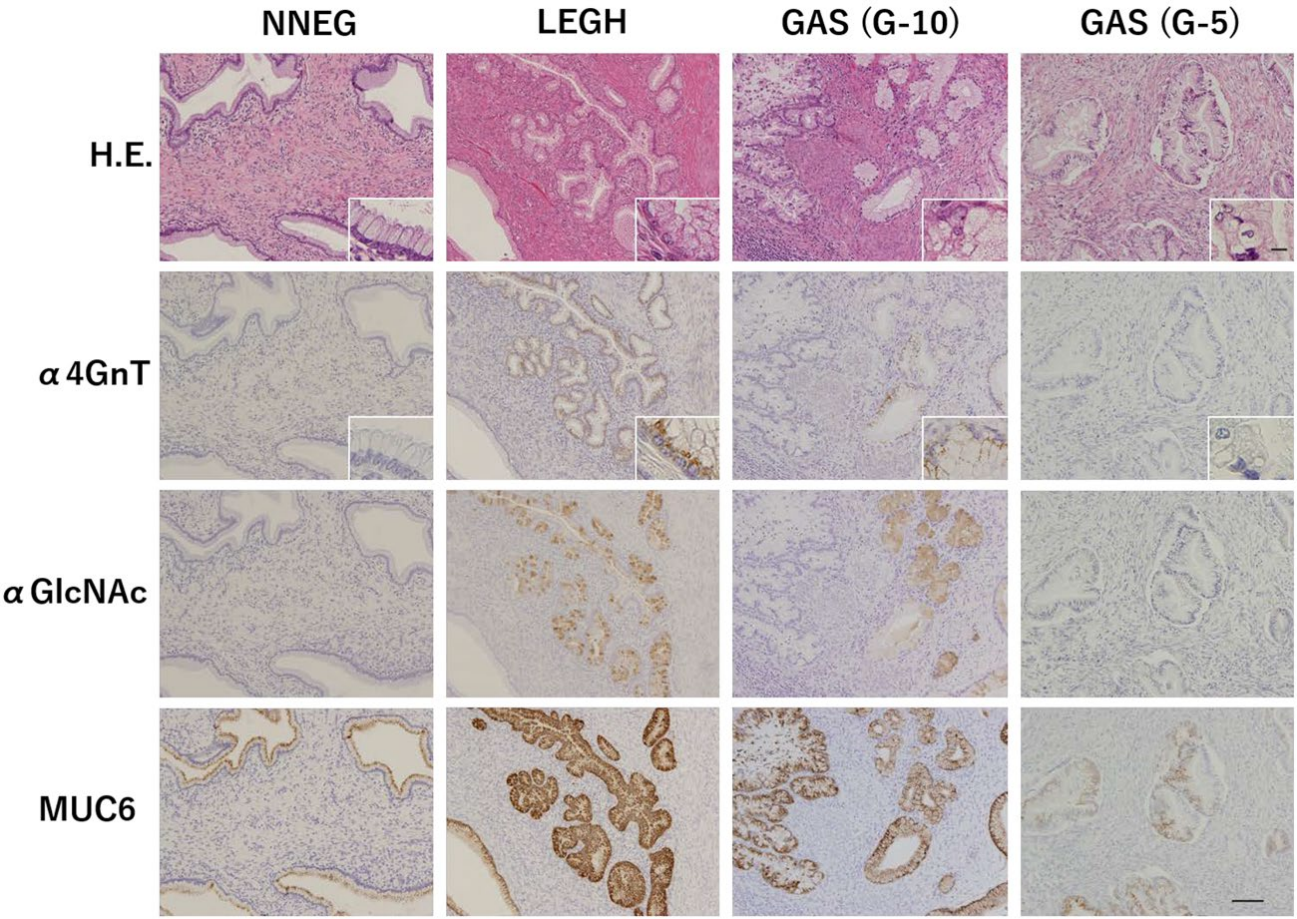
Immunohistochemical expression of α4GnT, αGlcNAc and MUC6 in NNEG, LEGH, and GAS (cases G-10 and G-5). Although α4GnT and αGlcNAc are expressed in LEGH, their expression decreases in GAS. Note that overall α4GnT and αGlcNAc are co-expressed in LEGH and GAS (case G-10), while MUC6 is expressed broadly in almost all histological types. Scale bar (bottom, right) = 100 μm. Insets show enlarged views of H.E. and α4GnT-stained sections. α4GnT in LEGH and GAS (case G-10) characteristically exhibits a “dot-like” pattern. Scale bar in inset (lower right) = 10 μm.

MUC6 was positively expressed in all 26 LEGH cases, 10 of 12 GAS cases and 7 of 11 NNEG cases (Table 1). The frequency of MUC6 positivity differed significantly between NNEG and LEGH (*P* < 0.01), but not between LEGH and GAS (*P* = 0.09) (Table 1). α4GnT-positive cells largely co-localized with αGlcNAc-positive cells in all positive lesions (Fig. 2). In LEGH, α4GnT and αGlcNAc were highly expressed in all 26 and 25 of 26 cases, respectively (Table 1). In GAS, α4GnT and αGlcNAc were co-expressed in the same 5 of 12 cases (Table 1). In NNEG, neither α4GnT nor αGlcNAc were detectable in any case (Fig. 2 and Table 1). Frequencies of α4GnT and αGlcNAc positivity differed significantly between NNEG and LEGH and between LEGH and GAS (*P* < 0.001) (Table 1).

**Table 1:** Frequency of positive cases for MUC6, αGlcNAc, α4GnT, and p16 expression in the NNEG, LEGH and GAS.

	Number of cases	MUC6 (%)	αGlcNAc (%)	α4GnT (%)	p16 (%)
NNEG	11	7 (63.6)*	0 (0.0)**	0 (0.0)**	0 (0.0)
LEGH	26	26 (100)*	25 (96.2)**	26 (100)**	0 (0.0)
GAS	12	10 (83.3)	5 (41.7)**	5 (41.7)**	0 (0.0)
Subtotal	49	43 (87.8)	30 (61.2)	31 (63.3)	0 (0.0)

*Significant difference in frequency of MUC6 positivity between NNEG and LEGH (*P* < 0.01) **Significant difference in frequency of αGlcNAc or α4GnT positivity between NNEG and LEGH (*P* < 0.001) and between LEGH and GAS (*P* < 0.001).

**Figure 3:**
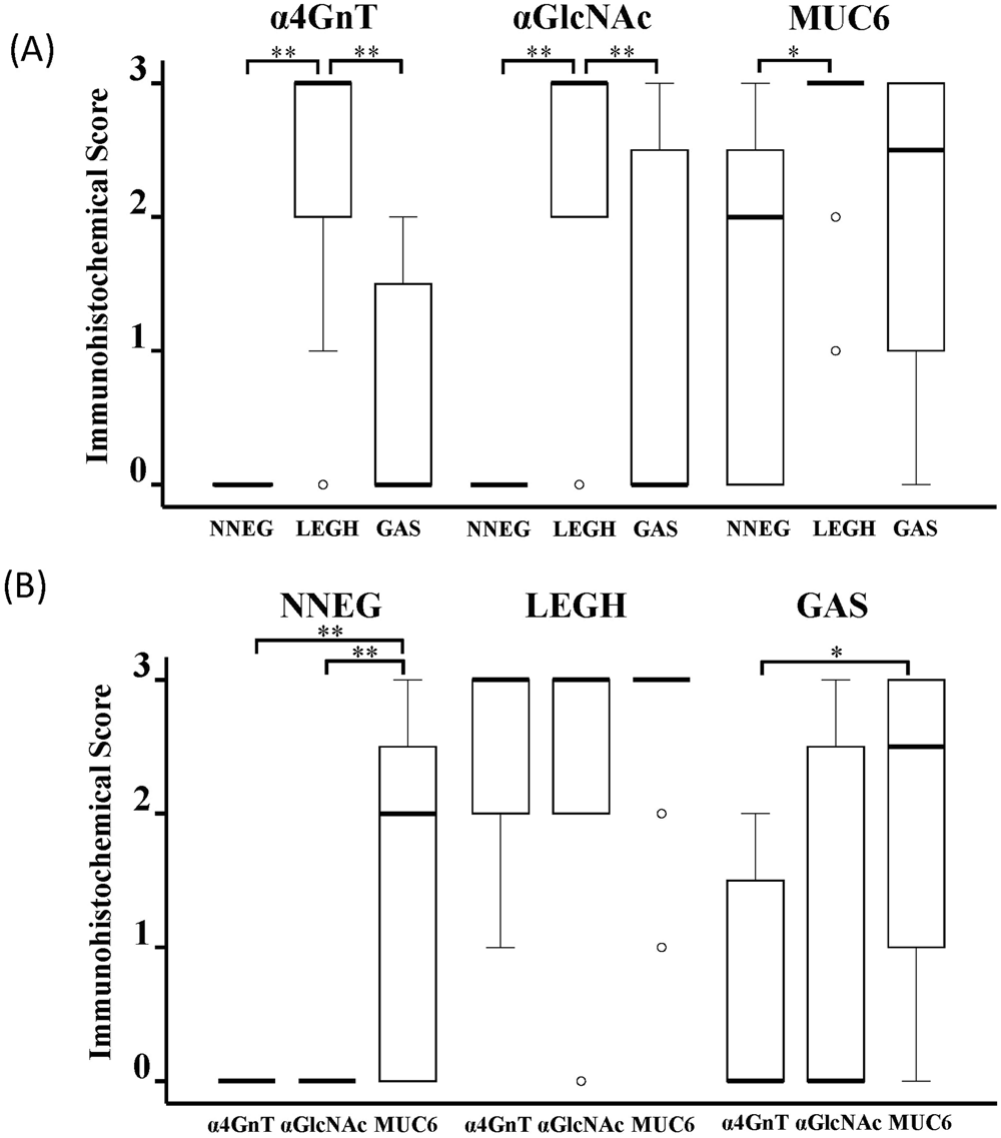
Semi-quantitation of α4GnT, αGlcNAc and MUC6 expression in NNEG, LEGH and GAS. The bisecting line, box boundaries, and whiskers indicate the median, 25th to 75th percentiles, and the estimated data range, respectively (A and B). (**A**) For each marker (αGlcNAc, α4GnT and MUC6), semi-quantified expression in NNEG vs LEGH vs GAS was compared. (**B**) In NNEG, LEGH and GAS, semi-quantified expression of 3 markers (αGlcNAc vs α4GnT vs MUC6) was compared. *P < 0.05, **P < 0.001 by Kruskal-Wallis test.

We next evaluated immunohistochemical scores from 0–3 (see Materials and Methods) of each marker including α4GnT, αGlcNAc and MUC6, in every case (Table S1) and compared differences among NNEG, LEGH and GAS histological types. The MUC6 score was high in all histological types: NNEG (median, 2.0 [interquartile range (IQR), 3.0]), LEGH (median, 3.0 [IQR, 0.0], and GAS (median, 2.5 [IQR, 2.0], respectively). The MUC6 score differed significantly between LEGH and NNEG (*P* < 0.05), but not between LEGH and GAS (*P* = 0.103) (Fig. 3A). By contrast, both α4GnT and αGlcNAc scores were consistently lower in NNEG (median, 0.0 [IQR, 1.75], either), GAS (median, 0.0 [IQR, 1.75], and median, 0.0 [IQR, 2.75], respectively) than in LEGH (median, 3.0 [IQR, 1.0], either). Statistically, both α4GnT and αGlcNAc scores in LEGH were significantly higher than those in NNEG (*P* < 0.001, either) in GAS (*P* = 0.01, either) (Fig. 3A).

We then compared these scores in each histological type (i.e., NNEG, LEGH and GAS). In NNEG cases, we observed significantly different α4GnT versus MUC6 as well as αGlcNAc versus MUC6 scores (*P* < 0.001 for NNEG) (Fig. 3B), differences not apparent in LEGH cases (Fig. 3B). In GAS cases, only the α4GnT and MUC6 scores showed a significant difference (*P* < 0.05) (Fig. 3B).

### Association of HPV infection in all 49 specimens

All NNEG, LEGH and GAS cases (49 cases) were p16 negative, which confirm that these 49 cases chosen for analysis were not derived from HPV infection (Table 1).

### Correlation between clinicopathological findings of GAS patients and α4GnT, αGlcNAc and MUC6 expression

In GAS patients, we did not observe significant differences in clinicopathological findings, such as patient age, FIGO stage, lymph node metastasis and ascites cytology, between cases positive or negative for MUC6, αGlcNAc and α4GnT markers (Table 2). However, both α4GnT- and αGlcNAc-positive cases consistently showed a lower frequency of lymph node metastasis and pelvic dissemination relative to negative cases (*P* = 0.175 for α4GnT, and *P* = 0.159 for αGlcNAc) (Table 2). Furthermore, the frequency of lymph node metastasis in cases scoring 2 or 3 for αGlcNAc expression was significantly lower than that in cases scored as 0 or 1 (*P* < 0.05) (Table S2).

**Table 2:** Clinicopathologic parameters of GAS patients relative to αGlcNAc, α4GnT and MUC6 expression.

	αGlcNAc		α4GnT		MUC6	
	positive/negative cases	*P*-value	positive/negative cases	*P*-value	positive/negative cases	*P*-value
Age at diagnosis						
≥55	2/4		2/4		6/0	
<55	3/3	0.500	3/3	0.500	4/2	0.227
FIGO Stage						
I-II	4/5		4/5		8/1	
III-IV	1/2	0.636	1/2	0.636	2/1	0.455
Metastasis to the lymphnode^†^						
Positive	1/4		1/4		4/1	
Negative	4/2	0.175	4/2	0.175	5/1	0.727
Ascitic cytology						
Positive	2/4		2/4		5/1	
Negative	3/3	0.500	3/3	0.500	5/1	0.773

^†^Lymphnode dissection not performed in one case.

GAS, gastric-type adenocarcinoma; FIGO, International Federation of Gynecology and Obstetrics.

### Correlation between GAS patients survival and α4GnT, αGlcNAc and MUC6 expression

Median follow-up period was 33 months. Median overall survival (OS) was 33 months (range from 3 to 163 months). Median progression free survival (PFS) was 13 months (range from 1 to 51 months). During our follow-up period, 2 of 12 GAS patients remained alive without cancer progression, while other 10 others died due to cancer progression.

**Figure 4:**
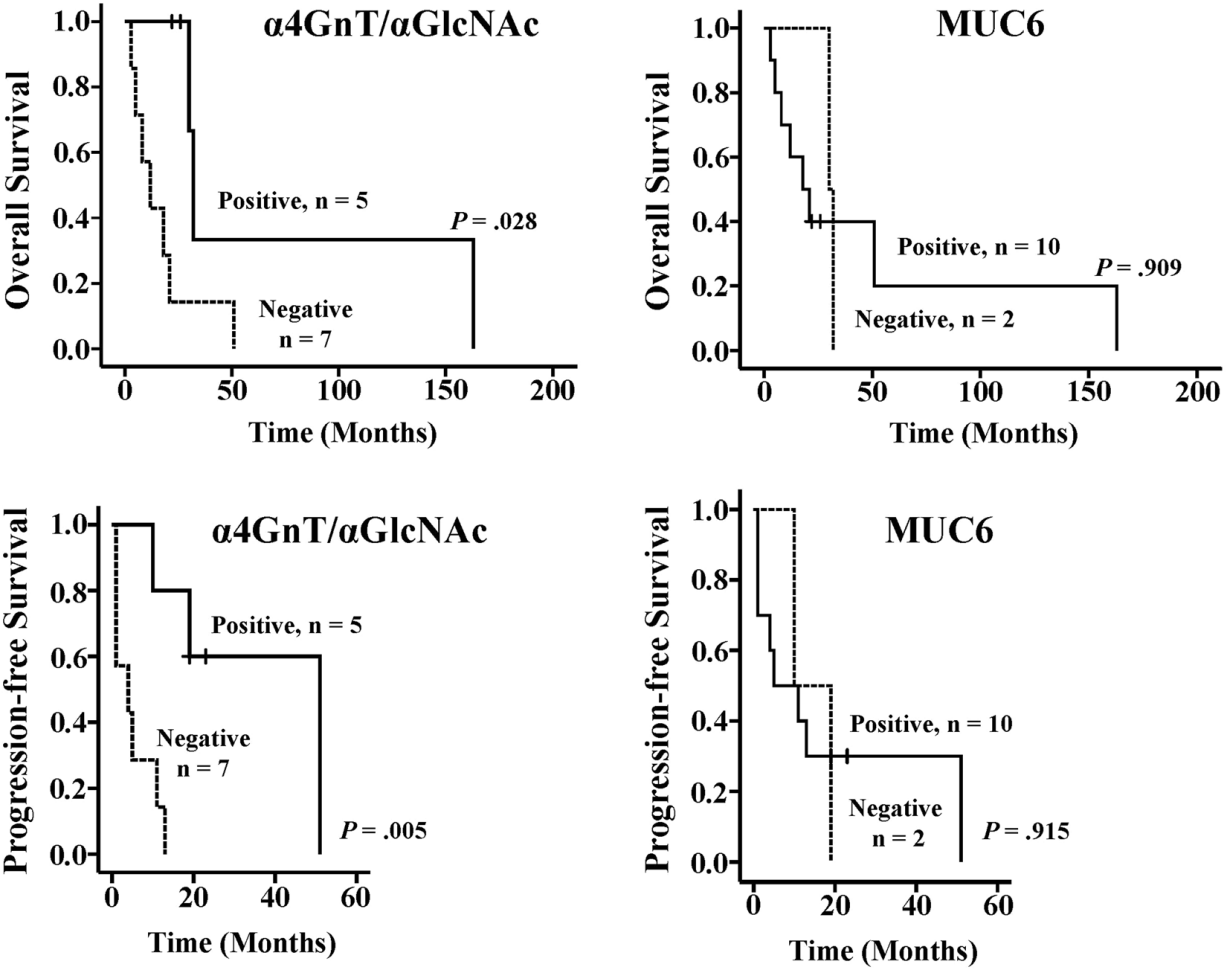
Overall survival (OS) and progression-free survival (PFS) curves of 12 GAS patients, based on phenotypic expression of α4GnT, αGlcNAc and MUC6 markers. Patients whose specimens are positive for α4GnT and αGlcNAc show more favorable prognosis (log-rank test, *P* < 0.05 for OS, *P* < 0.01 for PFS) (Left side figures). There are no significant differences in prognosis associated with differences in MUC6 expression (log-rank test, *P* = 0.909 for OS, *P* = 0.915 for PFS) (Right side figure).

In GAS patients, the estimated median survival of patients positive for both α4GnT and αGlcNAc (n = 5) was 30 months, whereas that of the patients negative for both (n = 7) was 12 months. In addition, the median progression-free survival period of α4GnT- and αGlcNAc-positive cases was 19 months, while that of α4GnT- and αGlcNAc-negative cases was 4 months. Thus, patients positive for α4GnT and αGlcNAc had significantly better prognosis relative to patients negative for both in OS rate and PFS (*P* < 0.05 for ΟS, and *P* < 0.01 for PFS) (Fig. 4). Relevant to MUC6 expression, we observed no significant differences in OS and PFS between positive and negative cases (*P* = 0.909 for OS, and *P* = 0.915 for PFS) (Fig. 4).

Lastly we evaluated the relationship between clinicopathological factors including the αGlcNAc and α4GnT expression and OS or PFS on 12 GAS cases. Univariate analysis demonstrated that only αGlcNAc and α4GnT expression was significantly correlated with OS (*P* = 0.045), and αGlcNAc and α4GnT expression and lymph node metastasis status were significantly correlated with PFS (*P* = 0.026 and 0.032, respectively) (Table 3). Variables with *P*-values less than 0.150 in the univariate analysis were included in the multivariate analysis. Multivariate analysis identified that αGlcNAc and α4GnT expression is the only independent prognostic factor in OS as well as in PFS. FIGO stage and lymph node metastasis status were found not to be independent prognostic factors in OS as well as in PFS (Table 3).

**Table 3:** Results of univariate and multivariate analysis of possible prognostic factors for GAS.

Factors	Univariate		Multivariate	
	HR (95% CI)	*P*	HR (95% CI)	*P*
Overall survival (n = 12^‡^)				
αGlcNAc/α4GnT Positive *vs* negative^†^	0.194 (0.039–0.964)	0.045*	0.126 (0.020–0.788)	0.027*
MUC6 Positive *vs* negative^†^	1.101 (0.213–5.695)	0.909		
FIGO stage I-II *vs* III-IV^†^	0.307 (0.067–1.414)	0.130	0.162 (0.025–1.049)	0.056
Age <55 *vs* ≥55^†^	0.351 (0.064–1.932)	0.229		
Lymphnode metastasis^‡^ Negative *vs* positive^†^	0.411 (0.097–1.750)	0.229		
Ascitic cytology Negative *vs* positive^†^	0.501 (0.026–0.958)	0.357		
Progression-free survival (n = 12^‡^)				
αGlcNAc/α4GnT Positive *vs* negative^†^	0.089 (0.010–0.752)	0.026*	0.093 (0.009–0.984)	0.048*
MUC6 Positive *vs* negative^†^	0.920 (0.189–4.470)	0.918		
FIGO stage I + II *vs* III + IV^†^	0.290 (0.058–1.455)	0.133	0.140 (0.018–1.092)	0.061
Age <55 *vs* ≥55^†^	0.884 (0.237–3.305)	0.855		
Lymphnode metastasis^‡^ Negative *vs* positive^†^	0.159 (0.030–0.854)	0.032*	0.284 (0.046–1.736)	0.173
Ascitic cytology Negative *vs* positive^†^	1.319 (0.352–4.935)	0.681		

^†^Reference. ^‡^In one case, lymph node dissection was not performed.

GAS, gastric-type adenocarcinoma; HR, hazard ratio; CI, confidence interval; FIGO, International Federation of Gynecology and Obstetrics. *P < 0.05.

## Discussion

Here, we report that α4GnT and αGlcNAc expression patterns are overall consistent, that is, they are absent in NNEG, acquired in LEGH, and then slightly decrease as carcinogenesis progression to GAS. Furthermore, decreased α4GnT and αGlcNAc expression was significantly and positively correlated with malignant prognosis in GAS patients. By contrast, MUC6 expression levels were relatively high throughout the sequence from NNEG to LEGH to GAS (Figs 2, 3B).

Previously, we isolated human cDNA encoding α4GnT, which catalyzes αGlcNAc biosynthesis by transferring GlcNAc from UDP-GlcNAc to terminal β-galactose residues present in *O*-glycans with an α1,4-linkage (Fig. 1)^26,27^. α4GnT protein is localized to the Golgi apparatus of gastric gland mucous cells, which corresponds to its expression as “dot-like” pattern (Fig. 2 and S1)^24^. Our study suggests that αGlcNAc biosynthesis is regulated by α4GnT expressed in cells of the uterine cervix, given that α4GnT-positive cells largely overlapped with αGlcNAc-positive cells in most cases (Fig. 2 and Table 1). Immunohistochemical αGlcNAc expression was often weak and difficult to be detected. On the other hand, α4GnT expression was always distinct with a typical supranuclear dot-like pattern (Fig. S1). Thus, α4GnT could serve as an alternative marker for αGlcNAc.

It is noteworthy that neither αGlcNAc nor α4GnT was detected in NNEG (Figs 2, 3 and Table 1). By contrast, MUC6 expression was often observed in NNEG (Figs 2, 3 and Table 1). Because LEGH histology resembles that of NNEG, differential diagnosis of the two is sometimes problematic^18^. Our findings indicate that evaluation of αGlcNAc or α4GnT could be helpful in differentiating LEGH from NNEG. We previously observed αGlcNAc, α4GnT and MUC6 expression in normal pyloric glands of human stomach^24^. Here, we observe that MUC6 but not α4GnT is frequently expressed in NNEG. In humans, chromosomal locations of MUC6 and α4GnT are 11p15.5 and 3q22.3, respectively, strongly suggesting that both genes are regulated separately^27,34^. It is possible that NNEG expressing MUC6 alone could be phenotypically regarded as an incomplete pyloric gland metaplasia-like lesion, while LEGH expressing both α4GnT/αGlcNAc and MUC6 could be seen as a complete pyloric gland metaplasia-like lesion. These findings are important not only for differential diagnosis of LEGH and NNEG but for our understanding of molecular mechanisms underlying pyloric gland metaplasia of uterine cervix.

We previously reported decreased αGlcNAc expression not only in cancer but in pre-malignant lesions of the human stomach, pancreas and uterine cervix^23,25,29^. In the latter, αGlcNAc expression decreases from typical LEGH to atypical LEGH or MDA^30^. These results indicate that decreased αGlcNAc expression is related to tumor progression from pre-malignant to a malignant status. Here, we extend these observations to predict malignant potential of an advanced cancer, in that αGlcNAc and α4GnT expression was significantly correlated with benign prognosis of GAS patients (Fig. 4). Furthermore, multivariate analysis demonstrated that αGlcNAc and α4GnT expression is an independent prognostic factor for GAS patients (Table 3). GAS is rare tumor, and number of GAS patients arising from LEGH is very small. Thus, further investigation will be of great significance to accumulate much more number of GAS patients to consolidate the prognostic significance of α4GnT/αGlcNAc expression as shown in the present study.

In conclusion, our work indicates that αGlcNAc catalyzed by α4GnT is relevant to two important developments in uterine cervix tumor, gastric type: one a positive correlation with the transition to LEGH from NNEG. The other a negative correlation with tumor progression from LEGH to GAS and unfavorable progression in GAS. However, molecular function of α4GnT in tumor progression remains to be clarified. Our immunohistochemical analysis of α4GnT and αGlcNAc expression in cervical resected specimens provides important tools for diagnosis of uterine cervical tumor, gastric type, and promotes understanding of tumor development. Both α4GnT and αGlcNAc are useful biomarkers for diagnosis of LEGH in uterine cervical biopsy specimens. Furthermore, decreased expression of α4GnT and αGlcNAc in follow-up biopsy of LEGH patients’ uterine cervix was closely associated with tumor progression to unfavorable GAS. Further studies will be of great significance to address molecular mechanisms underlying regulation of gastric type cervical tumor progression by α4GnT.

## Supplementary information

Table S1, S2

Figure S1
