# Supplementary material for: αGlcNAc and its catalyst α4GnT are diagnostic and prognostic markers in uterine cervical tumor, gastric type
Source: Sci Rep. 2019 Sep 10;9:13043. doi: 10.1038/s41598-019-49376-7 (PMC6737144; doi:10.1038/s41598-019-49376-7)
Supplement: Supplementary file 1 — Table S1, S2 [file 41598_2019_49376_MOESM1_ESM.pdf]

αGlcNAc and its catalyst α4GnT are diagnostic and prognostic markers in uterine cervical tumor, gastric type

Koichi Ida [1], Kazuhiro Yamanoi [2,3,4] Shiho Asaka [5], Hodaka Takeuchi [1], Tsutomu Miyamoto [1], Tanri Shiozawa [1] and Jun Nakayama [2,3]

[1] Department of Obstetrics and Gynecology, Shinshu University School of Medicine, Matsumoto 390-8621, Japan

[2] Department of Molecular Pathology, Shinshu University School of Medicine, Matsumoto 390-8621, Japan

[3] Institute for Biomedical Sciences, Interdisciplinary Cluster for Cutting Edge Research, Shinshu University, Matsumoto 390-8621, Japan

[4] Department of Pathology, Keio University School of Medicine, Tokyo 160-8582, Japan

[5] Department of Clinical Laboratory, Shinshu University Hospital, Matsumoto 390-8621, Japan

Corresponding author: Kazuhiro Yamanoi, MD, PhD, Department of Pathology, Keio University School of Medicine, 35 Shinanomachi, Shinjuku-ku, Tokyo 160-8582, Japan. Phone: +81-3-5363-3764; Fax: +81-3-3353-3290;

E-mail: yamanoi@keio.jp

List of all authors

Ida Koichi
Department of Obstetrics and Gynecology, Shinshu University School of Medicine, Matsumoto, Japan
tillafield@shinshu-u.ac.jp

Kazuhiro Yamanoi
Department of Molecular Pathology, Shinshu University School of Medicine, Matsumoto, Japan
kazyam@shinshu-u.ac.jp

Shiho Asaka
Department of Laboratory Medicine, Shinshu University Hospital, Matsumoto, Japan
ydash831@gmail.com

Hodaka Takeuchii
Department of Obstetrics and Gynecology, Shinshu University School of Medicine, Matsumoto, Japan
htakeuchi@shinshu-u.ac.jp

Tsutomu Miyamoto
Department of Obstetrics and Gynecology, Shinshu University School of Medicine, Matsumoto, Japan
tmiya@shinshu-u.ac.jp

Tanri Shiozawa
Department of Obstetrics and Gynecology, Shinshu University School of Medicine, Matsumoto, Japan
tanri@shinshu-u.ac.jp

Jun Nakayama
Department of Molecular Pathology, Shinshu University School of Medicine, Matsumoto, Japan
jnaka@shinshu-u.ac.jp

Table S1 Summary of immunohistochemical scores

NNEG				LEGH				GAS			
Case no.	α4GnT	αGlcNAc	MUC6	Case no.	α4GnT	αGlcNAc	MUC6	Case no.	α4GnT	αGlcNAc	MUC6
N-1	0	0	0	L-1	2	2	2	G-1	1	2	0
N-2	0	0	0	L-2	1	2	3	G-2	0	0	1
N-3	0	0	0	L-3	3	3	3	G-3	0	0	1
N-4	0	0	3	L-4	1	2	2	G-4	1	1	0
N-5	0	0	0	L-5	3	2	1	G-5	0	0	2
N-6	0	0	2	L-6	1	3	3	G-6	2	3	1
N-7	0	0	3	L-7	3	3	3	G-7	0	0	3
N-8	0	0	2	L-8	3	0	3	G-8	0	0	3
N-9	0	0	1	L-9	3	3	3	G-9	0	0	3
N-10	0	0	3	L-10	3	2	3	G-10	2	3	3
N-11	0	0	2	L-11	3	3	3	G-11	2	3	3
				L-12	3	3	3	G-12	0	0	3
				L-13	3	3	3				
				L-14	3	3	3				
				L-15	3	2	2				
				L-16	2	2	2				
				L-17	3	3	3				
				L-18	3	3	3				
				L-19	3	3	3				
				L-20	3	3	3				
				L-21	3	3	3				
				L-22	3	3	3				
				L-23	2	3	3				
				L-24	1	2	3				
				L-25	3	3	3				
				L-26	2	3	3				

Immunohistochemical scores ranged from 0 to 3: 0 (< 10% of positive cells), 1 (10-33% of positive cells), 2 (34-66% of positive cells) or 3, (≥ 67% of positive cells)

Table S2 Clinicopathologic Parameters of GAS and Relationships with expression of αGlcNAc, α4GnT and MUC6

	αGlcNAc		α4GnT		MUC6	
	score 3-2/1-0 cases	*P*-value	score 3-2/1-0 cases	*P*-value	score 3-2/1-0 cases	*P*-value
Age at diagnosis						
≥ 55	3/4		2/4		4/2	
< 55	1/3	.424	1/5	1.000	3/3	1.000
FIGO Stage						
I-II	4/5		3/6		6/3	
III-IV	0/3	.255	0/3	.382	1/2	.364
Metastasis to the lymph node†						
Positive	0/5		3/3		2/4	
Negative	4/2	.045*	0/5	.121	4/1	.175
Ascitic cytology						
Positive	2/4		2/4		4/2	
Negative	2/4	.727	1/5	.500	3/3	.500

†In one case, lymph node dissection was not performed.

GAS, gastric-type adenocarcinoma; FIGO, International Federation of Gynecology and Obstetrics.

* $P < .05$.
