# Supplementary material for: αGlcNAc and its catalyst α4GnT are diagnostic and prognostic markers in uterine cervical tumor, gastric type
Source: Sci Rep. 2019 Sep 10;9:13043. doi: 10.1038/s41598-019-49376-7 (PMC6737144; doi:10.1038/s41598-019-49376-7)
Supplement: Supplementary file 2 — Figure S1 [file 41598_2019_49376_MOESM2_ESM.pdf]

αGlcNAc and its catalyst α4GnT are diagnostic and prognostic markers in uterine cervical tumor, gastric type

Koichi Ida [1], Kazuhiro Yamanoi [2,3,4] Shiho Asaka [5], Hodaka Takeuchi [1], Tsutomu Miyamoto [1], Tanri Shiozawa [1] and Jun Nakayama [2,3]

[1] Department of Obstetrics and Gynecology, Shinshu University School of Medicine, Matsumoto 390-8621, Japan

[2] Department of Molecular Pathology, Shinshu University School of Medicine, Matsumoto 390-8621, Japan

[3] Institute for Biomedical Sciences, Interdisciplinary Cluster for Cutting Edge Research, Shinshu University, Matsumoto 390-8621, Japan

[4] Department of Pathology, Keio University School of Medicine, Tokyo 160-8582, Japan

[5] Department of Clinical Laboratory, Shinshu University Hospital, Matsumoto 390-8621, Japan

Corresponding author: Kazuhiro Yamanoi, MD, PhD, Department of Pathology, Keio University School of Medicine, 35 Shinanomachi, Shinjuku-ku, Tokyo 160-8582, Japan.

Phone: +81-3-5363-3764; Fax: +81-3-3353-3290;

E-mail: yamanoi@keio.jp

List of all authors

Ida Koichi
Department of Obstetrics and Gynecology, Shinshu University School of Medicine, Matsumoto, Japan
tillafield@shinshu-u.ac.jp

Kazuhiro Yamanoi
Department of Molecular Pathology, Shinshu University School of Medicine, Matsumoto, Japan
kazyam@shinshu-u.ac.jp

Shiho Asaka
Department of Laboratory Medicine, Shinshu University Hospital, Matsumoto, Japan
ydash831@gmail.com

Hodaka Takeuchii
Department of Obstetrics and Gynecology, Shinshu University School of Medicine, Matsumoto, Japan
htakeuchi@shinshu-u.ac.jp

Tsutomu Miyamoto
Department of Obstetrics and Gynecology, Shinshu University School of Medicine, Matsumoto, Japan
tmiya@shinshu-u.ac.jp

Tanri Shiozawa
Department of Obstetrics and Gynecology, Shinshu University School of Medicine, Matsumoto, Japan
tanri@shinshu-u.ac.jp

Jun Nakayama
Department of Molecular Pathology, Shinshu University School of Medicine, Matsumoto, Japan
jnaka@shinshu-u.ac.jp

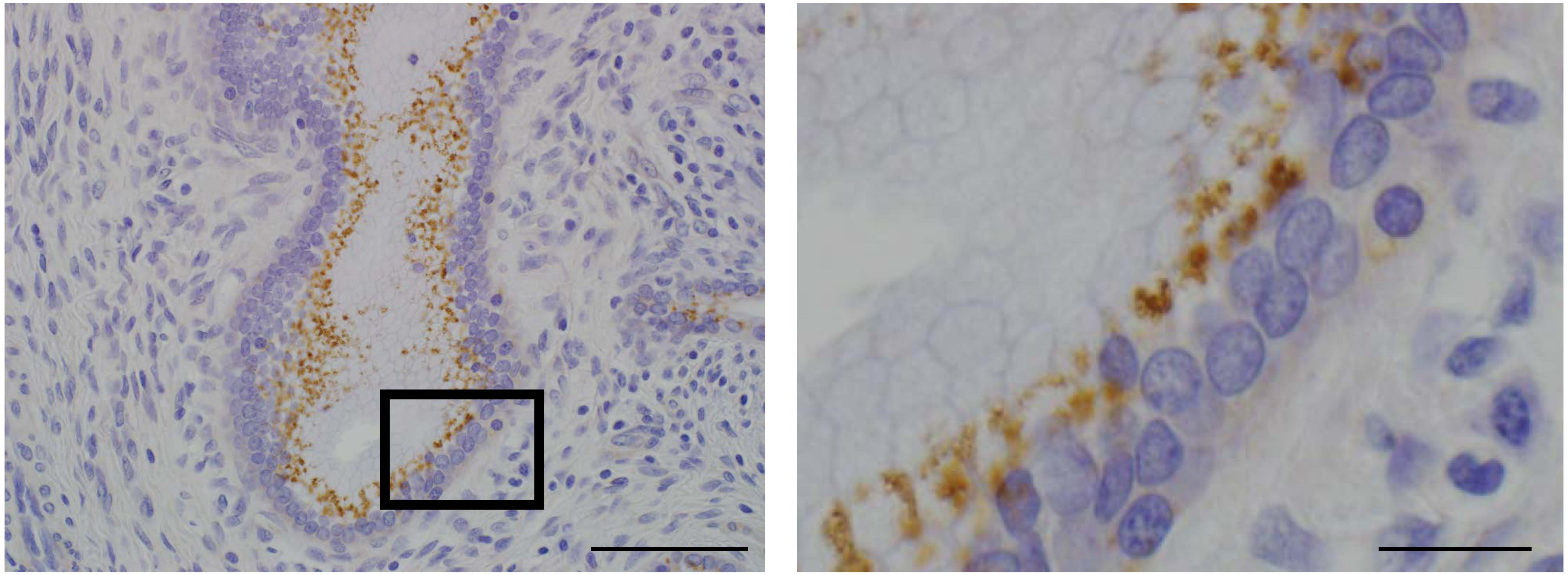

Fig. S1. Immunohistochemical expression of α4GnT in LEGH. Left figure shows low magnifiscent view, and right figure shows enlarged view of black bold box field in left figure. In right figure, brown dotted materials which indicate α4GnT "dot-like" expression are observed in each LEGH cells. Scale bar = 100μm (left figure), and 20μm (right figure)
